# Key predictors of psychological distress and wellbeing in Australian frontline healthcare workers during COVID-19 (Omicron wave)

**DOI:** 10.3389/fpsyg.2023.1200839

**Published:** 2023-07-07

**Authors:** Brian En Chyi Lee, Mathew Ling, Leanne Boyd, Craig A. Olsson, Jade Sheen

**Affiliations:** ^1^Centre for Social and Early Emotional Development, School of Psychology, Deakin University, Geelong, VIC, Australia; ^2^Neami National, Preston, VIC, Australia; ^3^Eastern Health, Box Hill, VIC, Australia; ^4^Department of Paediatrics, University of Melbourne, Parkville, VIC, Australia; ^5^Centre for Adolescent Health, Murdoch Children’s Research Institute, Parkville, VIC, Australia

**Keywords:** healthcare, doctors, nurses, COVID, pandemic, public and global mental health, risk factors, protective factors indent: first line: 1.27 cm formatted: normal

## Abstract

**Introduction:**

The COVID-19 pandemic has led to significant challenges for frontline healthcare workers’ (FHW), raising many mental health and wellbeing concerns for this cohort. To facilitate identification of risk and protective factors to inform treatment and interventions, this study investigated key predictors of psychological distress and subjective wellbeing in FHWs.

**Methods:**

During the Omicron wave of the COVID-19 pandemic (January 2022), Victorian (Australia) doctors, nurses, allied health and non-medical staff from Emergency Departments, Intensive Care units, Aged Care, Hospital In The Home, and COVID Wards completed a cross-sectional survey consisting of the Kessler 6 item (Psychological Distress), Personal Wellbeing Index (Subjective Wellbeing), Coronavirus Health Impact Survey tool (COVID-19 related factors) and occupational factors. Multivariable linear regressions were used to evaluate unadjusted and adjusted associations. Relative weight analysis was used to compare and identify key predictors.

**Results:**

Out of 167 participants, 18.1% screened positive for a probable mental illness and a further 15.3% screened positive for low wellbeing. Key risk factors for greater psychological distress included COVID infection worries, relationship stress and younger age. For both psychological distress and lower wellbeing, health status and supervisor support were key protective factors, while infection risks were key risk factors. Only positive changes in relationship quality was protective of lower wellbeing.

**Conclusion:**

This study highlights the significance of social determinants and individual level factors alongside work related factors, in influencing FHWs’ mental health and wellbeing during public health crises, such as the COVID-19 pandemic. Findings suggest that future interventions and supports should take a more holistic approach that considers work, social and individual level factors when supporting FHWs’ mental health and wellbeing.

## Introduction

Since the beginning of the COVID-19 pandemic, healthcare systems have been under significant pressure, leading to unprecedented challenges for healthcare workers worldwide. During this time, high rates of COVID-19 infections have led to concerning surges in hospital admissions (Verelst et al., 2020; Berger et al., 2022). This has translated into an increased and prolonged risk of COVID-19 infection among healthcare workers (Nguyen et al., 2020; Quigley et al., 2021; World Health Organisation, 2021), as well as a surge in their workloads (Billings et al., 2021; Spányik et al., 2022) and a complete change in their working procedures (Digby et al., 2021; Hunt et al., 2022). Under these conditions, healthcare workers have been working under significant challenges, both physically and mentally.

Existing research suggests that working in hospitals during the pandemic have contributed to high rates of burnout (Magnavita et al., 2021), insomnia (Salari et al., 2020; Sahebi et al., 2021; Lee et al., 2023) and psychological distress. High prevalence of mental health disorders have also been documented in this cohort, such as depression (Yan et al., 2021; Lee et al., 2023), anxiety (Raoofi et al., 2021; Yan et al., 2021; Lee et al., 2023), and PTSD (Yan et al., 2021; Lee et al., 2023). Emerging evidence also links healthcare roles during the pandemic with a deterioration in overall wellbeing, suggesting that COVID-19 impacts on healthcare workers have spanned across multiple life domains during this time (McGuinness et al., 2022). This is concerning as COVID-19 outbreaks are persisting and continue to escalate the stress on healthcare systems worldwide (World Health Organisation, 2022), placing healthcare workers at high risk of continued and cumulative impacts on their mental health and wellbeing.

Healthcare workers working in frontline settings with high-risk COVID-19 infections may be particularly vulnerable, such as those working in emergency departments, intensive care units and COVID wards, as proximity to risk during disasters can significantly increase an individual’s vulnerability to mental illness (May and Wisco, 2016). Studies of coronavirus outbreaks support this contention, highlighting that healthcare workers directly caring for infected patients in frontline settings have high risks of mental health impacts (De Brier et al., 2020; Kisely et al., 2020; Muller et al., 2020). These findings were also consistent during the COVID-19 pandemic, with studies demonstrating that frontline healthcare workers (FHWs) experienced more anxiety, depression, and traumatic stress than their non-frontline counterparts (Lai et al., 2020; Wang et al., 2020; Kim and Lee, 2022). Long-term mental health consequences are also a concern as findings on past coronavirus outbreaks suggest that some FHWs can be affected up to 2 years after outbreaks (Liu et al., 2012; Galli et al., 2020; Chau et al., 2021). Given these findings, there is a clear need to identify FHWs at greatest risk and key predictors of their psychological distress and wellbeing to inform interventions and supports that are critical during the COVID-19 pandemic and future infectious disease outbreaks.

Most studies strongly support that work-related stressors are key predictors of psychological distress in FHWs during the COVID-19 pandemic, such as work-related infection risks, level of work experience and organisational support (De Brier et al., 2020; Kisely et al., 2020; al Falasi et al., 2021). Thus, recommendations and interventions for FHWs to date have largely revolved around workplace supports and enhancing infection control procedures. However, the impacts of the COVID-19 pandemic have been wide-ranging, and few have investigated other important determinants of FHWs’ mental health beyond work, such as social and pandemic related determinants. Studies on the general community have shown that pandemic and social stressors can also lead to significantly greater distress and lower wellbeing. These stressors include lockdowns (Westrupp et al., 2021a,b), community infection risks (Fitzpatrick et al., 2020; Kim et al., 2022) and relational impacts (Cassinat et al., 2021; Sheen et al., 2021; Wang et al., 2021). Emerging qualitative evidence also support these findings in FHWs, showing that these social stressors have placed significant burden on FHWs during the COVID-19 pandemic (Schaffer et al., 2022; Sheen et al., 2022). These findings, therefore, highlight the need to further our understanding of key predictors of psychological distress and wellbeing amongst FHWs during the COVID-19 pandemic that considers stressors beyond work, such as pandemic and social stressors.

Nevertheless, studies have largely been conducted early in the pandemic and there is currently a dearth of research on more recent waves of COVID-19 outbreaks, such as the Omicron wave. Updated findings is thus necessary, especially when the extended nature of the COVID-19 pandemic places FHWs at high risk of continued and cumulative mental health and wellbeing impacts. Continued understanding and investigation into key predictors of FHWs’ psychological distress and wellbeing during the COVID-19 pandemic is critical to inform and enhance the effectiveness of future interventions and facilitate targeted interventions for highly vulnerable FHWs, during the COVID-19 pandemic and in future infectious disease outbreaks.

### Research aims

This study aimed to investigate key predictors of psychological distress and subjective wellbeing among FHWs during the COVID-19 pandemic. Specifically, this study aimed to examine to what extent did demographics, health factors, COVID-infection factors, occupational factors, lockdown stressors and relational factors have an effect on psychological distress and subjective wellbeing in FHWs during the COVID-19 pandemic.

## Methods

This study followed and adhered to the reporting guidelines of the STROBE guidelines for cross-sectional studies (Supplementary File S1).

### Study design

This study used cross-sectional data collected in the third timepoint of a longitudinal cohort study on FHWs’ working in Victoria, Australia. The data used in this study was collected between late January 2022 to early March 2022 using an online survey, administered on RedCap.

### Recruitment

Participants were recruited state-wide in Victoria (Australia) from a large metropolitan health service (Eastern Health), and five major Australian healthcare associations: Australian medical association Vic (AMAVic), Australian Nursing and Midwifery Federation Vic (ANMFVic), Aged & Community Care Providers Association (ACCPA), Victorian Healthcare Association (VHA) and Health Services Union (HSU). Participants were eligible to participate if they worked in any capacity in Victorian (Australia) hospitals and in any of the following departments: the Emergency Departments, Intensive Care Units, COVID Wards, Hospital in the Home or Aged Care. These departments were chosen to represent frontline healthcare working sites and to recruit FHWs, as they posed the highest risk of COVID-19 infection in Victoria, Australia.

### Ethics

Study and ethics approval (HEAG 2020-296) was obtained from Deakin University High Risk Ethics Committee, Eastern Health’s Ethics Committee, and all partnering healthcare associations prior to the commencement of recruitment and data collection. Participants provided their consent and voluntarily participated. To ensure confidentiality, participant’s data were all de-identified prior to data-analysis.

### Context

During the data collection period, the general community were experiencing the easing of COVID-19 restrictions in Victoria, Australia (Premier of Victoria, 2022a). However, Victoria (Australia) was just coming out of the Omicron wave and largest ever surge of positive COVID-19 cases and hospitalisation since the start of the COVID-19 pandemic, which started in November and peaked in mid-January 2022 (State Government of Victoria, 2023). As such, the Victorian government declared a code brown emergency due to the influx of COVID-19 patients in hospitals (Premier of Victoria, 2022b). This meant that hospitals could configure or shutdown non-essential services to free staff, redeploy staff to higher priority departments, and ask staff to return from leave.

### Measures

The survey collected data using individual items and scales from the Coronavirus Health Impact Survey tool (CRISIS) (Nikolaidis et al., 2021). Individual CRISIS items used in the survey included two *demographic* items: age and gender, two *health* related items: self-reported physical health and pre-COVID-19 mental health, two *lockdown* related items: self-reported stress with lockdown restrictions and time spent outdoors, and three *COVID-infection* related items: self-reported risk of infection from work, risk of infection from the community, and COVID-19 diagnosis.

A modified version of the *COVID-19 infection worries* scale from the CRISIS tool was also used in the survey. *COVID-19 infection worries* was measured using a validated scale score of four items measuring to what extent participants were worried about (1) being infected, (2) their family being infected, and the impacts of infection on their (3) mental health and (4) physical health. The original scale was found to have high internally consistency with a coefficient Omega of >0.8 and good unidimensionality (CFI > 0.95) (Nikolaidis et al., 2021). In our study, to reflect worries of infection more accurately, we removed the items that related to reading and talking about COVID-19, and hope that the pandemic will end soon. Our modified version had good internal consistency (McDonald’s ω = 0.88) and unidimensionality (CFI = 0.96).

To measure relational factors, two items from the CRISIS tool measuring (1) *changes in relationship quality* with regards to family and (2) *changes in relationship quality* with regards to social contacts were combined to avoid multi-collinearity in regression models and derive a single scale score of overall *changes in relationship quality*. This scale showed moderate internal consistency (McDonald’s ω = 0.60). Overall *relationship stress* was measured using a single scale score derived from the mean of two items measuring (1) *relationship stress* with regards to family and (2) *relationship stress* with regards to friends (5-point Likert scale, 1 = Not at all to 5 = Extremely). This scale showed good internal consistency (McDonald’s ω = 0.76).

To measure occupational factors, participants were asked to specify their occupation, whether they provided direct care to COVID-19 patients in the last 2 weeks, and a self-report rating of their supervisor’s support for mental health and wellbeing during the COVID-19 pandemic.

To measure psychological distress participants responded on a Likert scale from 1 (none of the time) to 4 (all of the time) to items on the Kessler Psychological Distress Scale- Six item (K6) scale, which has good internal consistency and reliability with a Cronbach’s alpha of 0.89 (Kessler et al., 2003). In this study Cronbach’s alpha and McDonald’s Omega was both 0.88 and CFI was 0.95 showing good internal consistency and unidimensionality. Scores were summed to produce a final score. A cut-point of 19 was used to indicate the presence of a probable mental health disorder. This cut-point has been shown to have high specificity (96%) but lower sensitivity (36%) in detecting health disorder diagnosed through clinical interviews (Kessler et al., 2003). The Kessler Psychological Distress Scale has also shown to have good measurement invariance across different age groups (Sunderland et al., 2013) and gender (Drapeau et al., 2010), as well being validated across a wide range of populations from different countries and culture (Donker et al., 2010; Oakley Browne et al., 2010; Andersen et al., 2011; Fernandes et al., 2011; Bu et al., 2017), including Australia (Slade et al., 2011).

To measure Subjective wellbeing participants completed the Personal Wellbeing Index (PWI), which has shown to have good internal consistency and reliability with a Cronbach’s alpha between 0.7 to 0.85 in the Australian population. Test–retest reliability over 1 to 2 weeks have shown to be good with an intra-class correlation of 0.84. In this study, internal consistency (Cronbach’s alpha = 0.92, McDonald’s Omega = 0.92) and unidimensionality of the PWI was good (CFI = 0.98). The PWI has seven domains that consistently form one factor, explaining 50% of the variance in the domain “satisfaction as a whole” in the Australian population (International Wellbeing Group, 2013). The seven domains are measured on a Likert scale from 0 (no satisfaction at all) to 10 (completely satisfied), which were totaled, averaged, and multiplied by 10 to produce the single mean PWI score. The PWI has been validated in Australia (International Wellbeing Group, 2013) and across a wide range of countries and cultures, showing good measurement invariance (Żemojtel-Piotrowska et al., 2017; Jovanović et al., 2019).

### Data analysis procedure

To address missing values observed in the data, missing data were multiply imputed in R using the MICE package (van Buuren and Groothuis-Oudshoorn, 2011). Fifty imputations were conducted and 5 iterations, which was conservative given that missingness did not exceed 11%. To assist imputations, auxiliary variables were used in the imputation. Outcome variables (i.e., K6 and PWI total scores) were not imputed and were only computed after imputation to reduce any potential bias during the imputation process. All analyses were conducted and pooled using Rubin’s rule (Rubin, 2004).

T-tests were used to compare sample means with normative data that was nationally representative of the Australian population and collected at similar timepoints as the current study. Normative data was taken from the Australian Unity Wellbeing Index national 2022 report (Khor et al., 2022) for PWI scores and the Australian National University national COVID-19 tracking poll (Biddle and Gray, 2022) for K6 scores. Univariate associations between predictors and outcome variables were analysed using unadjusted linear regressions.

Multivariable linear regression was used to estimate the total effect (i.e., includes both the direct and indirect effects) of each predictor on psychological distress and subjective wellbeing while adjusting for covariates. For each predictor, a minimally sufficient adjustment set (MSAS) was identified. MSAS is the minimum set of covariates to adjust in models to estimate the total effect and avoid distorted and biasing associations that can occur in typical regression models where covariate adjustment is based only on significance of results (Rohrer, 2018; Griffith et al., 2020). To identify MSAS for each predicter, directed acyclic graphs (DAG) were developed (Rohrer, 2018), using DAGitty^1^ (Textor et al., 2016). The DAG is a graph that represents a theoretical framework around the causal relationships between variables, which is represented by arrows that are “directed” and imply a casual sequence. Once the theoretical framework was developed, Daggity applied the Pearl’s single and back door criterion (Pearl, 2009; Rohrer, 2018) to find the MSAS for each predictor’s model. When developing the DAG, the authors followed current recommendations (Tennant et al., 2021) and to ensure that omitted relationships were justified, all assumed relationships were tested for conditional independence in the data using polychoric correlations with the Lavaan package (Rosseel, 2012) in R based on current protocols (Ankan et al., 2021). The final relationships assumed in our DAG are presented in the supplements (Supplementary File S2). Multivariate linear regressions were then conducted individually, controlling for each predictors’ MSAS (see Table 1 for MSAS for each predictor).

**Table 1:** Minimum sufficient adjusted sets (MSAS) for each predictor examined in each regression models.

Predictor variables	MSAS*
Age	N/A
Gender	N/A
Pre-COVID mental health	Age, occupation, gender
Physical health	Age, COVID diagnosis, Pre-COVID mental health, occupation, gender, supervisor support
Occupation	Age, gender
Direct care	Age, occupation, gender
Supervisor support	Age, direct care, pre-COVID mental health, occupation, gender
Community infection risk	Age, COVID diagnosis, pre-COVID mental health, physical health, gender
Work infection risk	Age, community infection risk, COVID diagnosis, direct care, pre-COVID mental health, occupation, physical health, gender, supervisor support
COVID-19 infection worries	Age, community infection risk, COVID diagnosis, direct care, pre-COVID mental health, occupation, physical health, gender, supervisor support, work infection risk
COVID diagnosis	Age, direct care, gender, supervisor support
Outdoors time	COVID infection worries, age, community infection risk, COVID diagnosis, pre-COVID mental health, physical health, gender, work infection risk
Stress from lockdown restrictions	COVID infection worries, age, community infection risk, COVID diagnosis, pre-COVID mental health, occupation, physical health, changes in relationship quality, gender, supervisor support, work infection risk
Relationship stress	COVID infection worries, age, community infection risk, COVID diagnosis, pre-COVID mental health, outdoors time, physical health, changes in relationship quality, gender, stress from lockdown restrictions, supervisor support, work infection risk
Changes in relationship quality	COVID infection worries, age, community infection risk, COVID diagnosis, pre-COVID mental health, outdoors time, physical health, gender, supervisor support, work infection risk

*Minimally sufficient adjustment set: the minimum number of covariates needed to identify the total effect without inducing biasing associations.

Regression models were tested for heteroscedasticity using Breusch-Pagan test. Only subjective wellbeing models were found to be significantly heteroscedastic. Thus, utilising the sandwich package in R (Zeileis, 2004; Zeileis et al., 2020), Heteroscedasticity-Consistent (HC) standard errors, specifically the recommended HC3 estimator (Long and Ervin, 2000), were used in subjective wellbeing regression models. Using Gpower 3.1, *post hoc* power analysis showed that at alpha level 0.05, the sample size was large enough to detect statistical significance in effects ( $R^{2}$ ) above 4.7% for psychological distress and 4.6% for subjective wellbeing.

To identify key predictors and compare the unique contribution of each predictors’ total effect on K6 and PWI scores, relative importance analysis was conducted using relaimpo R package using the Lindemann-Merenda-Gold (LMG) method (Groemping, 2006). This analysis partitions variance explained in outcome variables by averaging over orderings and accounting for intercorrelations among covariates. This produces an unbiased $R^{2}$ for individual predictors that is decomposed and adjusts for other covariates, which is not typically accounted for in common estimates of decomposed $R^{2}$ that are biased by multi-collinearity (Groemping, 2006; Tonidandel and LeBreton, 2011).

Significance thresholds were all set at 0.05 for all analyses. Confidence intervals (95% CI) were computed by bootstrapping based on 1,000 bootstrap resamples from each of the 50 imputed datasets in R (Schomaker and Heumann, 2018) and pooling them together using Rubin’s rule (Rubin, 2004).

## Results

### Descriptive statistics

One hundred and seventy-two Victorian frontline healthcare workers completed the survey. Two participants that noted “other” as their gender and three responses that did not identify their gender were removed due to extreme uneven distribution in analyses with gender as a predictor, leaving a total of 167 responses. Details of participants’ characteristics are displayed in Table 2. In brief, the majority of respondents were female (88.6%), nurses and midwives (70.1%), and working in ICU (32.9%). When compared to previous data obtained from a large state-wide health service (Holton et al., 2020), the sample shows a good representation of the nursing population but an overrepresentation of allied health staff and under representation of physicians. Most respondents worked in public hospitals (92.3%). Only 17.4% of participants had been diagnosed with COVID-19 since the start of the pandemic. Approximately half of participants (55.1%) worked directly with COVID-19 patients.

**Table 2:** Participant characteristics.

	*n* (%)
Total sample	167 (100%)*
Age	Mean (SD) = 42.2 (11.3)
**Gender**	
Male	19 (11.4%)
Female	148 (88.6%)
**Occupation**	
Physician	16 (9.6%)
Nurse and midwives	117 (70.1%)
Allied health	25 (15.0%)
Non-medical staff	9 (5.4%)
**Ward**	
ICU	55 (32.9%)
ED	45 (26.9%)
COVID related wards	27 (16.2%)
Hospital in the home	11 (6.6%)
Aged care	21 (12.6%)
Multiple departments	8 (4.8%)
**Private or public hospital**	
Private	11 (7.7%)
Public	131 (92.3%)
**Covid diagnosis**	
No	138 (82.6%)
Yes	29 (17.4%)
**Provided direct care to COVID patients**	
No	75 (44.9%)
Yes	92 (55.1%)
**K6**	
≤19	131 (81.9%)
≥19	29 (18.1%)
**PWI**	
≥70	85 (52.1%)
50–70	53 (31.5%)
≤50	25 (15.3%)

* = Not all values sum to total due to missingness.

The K6 measure was completed by 161 participants. Mean psychological distress among participants was 13.70 (SD = 4.96), which was significantly higher (t = 5.4, *p* < 0.001) compared to normative data (Normative mean = 11.6) that was collected during the same period as this study (Biddle and Gray, 2022). Using the recommended cut off of 19, 18.1% of the sample screened positive for a probable mental health disorder.

**Figure 1:**
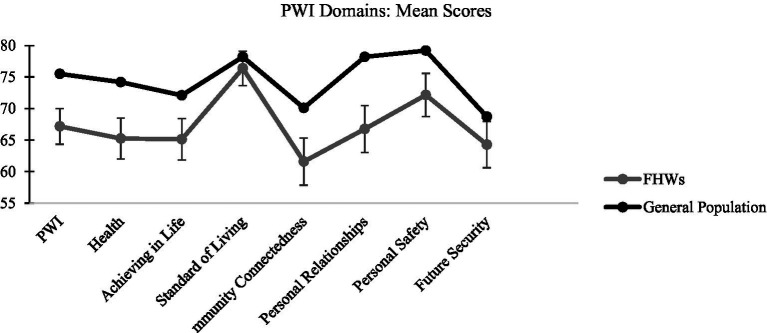
Means and standard deviations for FHWs in this study and the Australian Unity Wellbeing Index Norm on the PWI and sub-domains.

The PWI measure was completed by 163 participants. Mean SWI was 67.17 (SD = 18.22), significantly lower (p < 0.001) than aggregated normative data (normative mean = 75.5; Khor et al., 2022). Overall, 25 participants had SWI mean scores lower than 50, indicating that 15.3% of participants were experiencing concerningly low levels of wellbeing. Sample means were also consistently significantly lower (*p* < 0.05) than normative data in all sub-domains except for standard of living (*p* < 0.05; Figure 1). Based on Cohens’ D effect sizes, these significant mean differences were small (Cohens’ D: 0.2–0.5) for all sub-domains except for future security (Cohens’ D < 0.2), which was negligible.

### Regression and relative importance analysis-Psychological distress (K6) and Regression and relative importance analysis-Subjective wellbeing (PWI)

The results for the adjusted and unadjusted regression analyses, and relative importance analysis for psychological distress are displayed in Table 3. In the multivariate models adjusting for the MSAS, significant total effects were found for age, both health factors, supervisor support, all COVID infection related factors and relationship stress. Results show that younger age, higher perceived work infection risk, high perceived community infection risk, more COVID infection worries, a previous positive COVID-diagnosis and more relationship stress were significant risk factors and associated with higher levels of psychological distress. Better supervisor support, better pre-covid mental health, and better physical health were found to be protective and significantly associated with lower psychological distress.

**Table 3:** Results of the unadjusted (univariate) and adjusted (multivariate) regressions for psychological distress.

	Psychological distress				
	Unadjusted	Adjusted			
	B	B	LL	UL	*R*^2^
Age	−0.11**	−0.11**	−0.18	−0.05	6.67%
Gender (ref = male)	1.84	1.84	−0.37	4.05	1.31%
Pre-COVID mental health	−1.13**	−1.21**	−2.11	−0.32	5.09%
Physical health	−1.75***	−1.15**	−2.11	−0.18	6.99%
**Occupation (ref = nurses)**					
Allied health	−1.16	−1.64	−3.74	0.46	3.38%
Non-med	−3.45	−3.11	−6.74	0.53	3.38%
Physicians	−1.89	−1.66	−4.31	0.98	3.38%
Supervisor support	−1.15***	−1.06***	−1.69	−0.43	7.88%
Direct care (ref = no direct care)	0.51	−0.02	−1.57	1.53	0.11%
Work infection risk	1.51***	0.90**	0.19	1.61	6.21%
Community infection risk	1.28**	1.14**	0.44	1.83	6.15%
COVID-19 infection worries	2.57***	1.79***	0.87	2.70	17.66%
COVID diagnosis (ref = no diagnosis)	2.29*	2.14*	0.08	4.20	2.76%
Outdoors’s time	−1.15***	−0.51	−1.10	0.08	4.21%
Stress from lockdown restrictions	1.78***	0.68	−0.33	1.69	3.83%
Relationship stress	2.15***	0.97**	0.22	1.71	8.63%
Changes in relationship quality	−1.40*	−0.62	−1.52	0.28	1.68%

* = *p* < 0.05, ** = *p* < 0.01, *** = *p* < 0.001, LL = lower limit of bootstrapped 95%CI, UL = upper limit of bootstrapped 95%CI.

**Figure 2:**
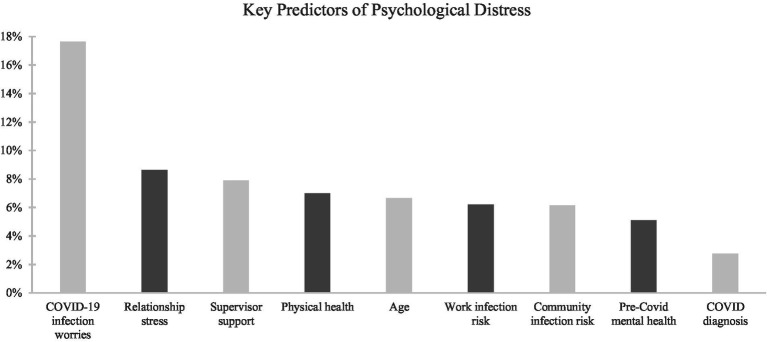
Relative importance analysis-psychological distress.

Based on the results of the relative importance analysis, COVID worries ( $R^{2}$ =17.66%) explained the most unique variance in psychological distress and was considered a large effect based on Cohen’s proposed magnitude for $R^{2}$ effect sizes. All other significant predictors explained moderate amounts of unique variance in psychological distress, $R^{2}$ ranging from 2.76 to 8.63% (Predictor ranking displayed in Figure 2).

### Regression and relative importance analysis-PWI

The results for the PWI and its sub-domains’ regression analyses are presented in Tables 4–6. After adjusting for the MSAS, significant total effects on PWI were found for both health factors, occupation, supervisor support, work infection risk and changes in relationship quality. Results show that a higher rating of pre-COVID mental health, physical health and supervisor support were all significantly associated with a higher overall subjective wellbeing. Positive changes in relationship quality were also associated with a higher subjective wellbeing. Being a nurse was associated with a lower subjective wellbeing, however, only when compared to allied health staff. Higher perceived work infection risk was associated with lower subjective wellbeing. When analysed within the PWI sub-domains, pre-COVID mental health, physical health and supervisor support were consistently significant predictors in all domains except for personal relationships, where supervisor support had no significant effect. In terms of occupation, being a nurse was associated with a lower satisfaction in standard of living, and future security when compared to allied health, and a lower satisfaction of health when compared to non-medical staff. Nurses also had lower satisfaction in their achievement in life when compared to physicians. Work infection risk was only a significant predictor of standard of living, personal safety, and future security. Relationship quality was only predictive of standard of living, personal relationships, and community connectedness.

**Table 4:** Results of the unadjusted (univariate) and adjusted (multivariate) regressions for PWI, standard of living, and health domains.

	Personal wellbeing index					Standard of living					Health				
	Unadjusted	Adjusted				Unadjusted	Adjusted				Unadjusted	Adjusted			
	B	B	LL	UL	*R*^2^	B	B	LL	UL	*R*^2^	B	B	LL	UL	*R*^2^
Age	0.08	0.08	−0.16	0.31	0.22%	0.14	0.14	−0.09	0.36	0.73%	0.07	0.07	−0.22	0.35	0.12%
Gender (ref = male)	−4.47	−4.47	−11.46	2.51	0.57%	−0.24	−0.24	−11.58	11.11	0.00%	−5.55	−5.55	−14.79	3.69	0.64%
Pre-COVID mental health	6.45***	6.48***	3.06	9.90	11.41%	5.26**	5.28**	1.94	8.62	7.83%	6.98***	7.18***	3.38	10.98	9.98%
Physical health	10.28***	8.35***	4.70	12.00	20.60%	7.8***	5.77***	2.60	8.94	11.55%	13.6***	12.64***	7.88	17.40	29.28%
**Occupation (ref = nurses)**															
Allied health	6.69*	7.28*	0.12	14.44	2.67%	7.09*	7.57*	0.60	14.55	3.07%	6.39	7.00	−1.53	15.54	2.61%
Non-med	1.62	1.16	−9.97	12.30	2.67%	−3.8	−4.37	−15.28	6.54	3.07%	10.53*	10.12*	1.31	18.93	2.61%
Physicians	7.49	6.49	−2.31	15.29	2.67%	5.09	5.79	−5.93	17.52	3.07%	7.19	5.6	−7.59	18.78	2.61%
Supervisor support	3.88***	3.37***	1.34	5.39	6.61%	3.77***	3.12***	1.38	4.86	6.21%	3.24**	2.9*	0.46	5.34	3.45%
Direct care (ref = no direct care)	−2.92	−2.27	−7.94	3.41	0.51%	−6.36*	−6.04*	−11.30	−0.78	2.92%	0.06	1.42	−5.30	8.14	0.05%
Work infection risk	−4.05**	−2.52*	−4.87	−0.17	3.32%	−4.64***	−2.85*	−5.29	−0.40	4.60%	−2.46	−1.5	−4.03	1.03	0.79%
Community infection risk	−1.9	−2.49	−5.26	0.28	1.63%	−1.92	−2.46	−5.26	0.35	1.62%	−1.8	−2.3	−5.09	0.50	1.11%
COVID-19 infection worries	−4.31**	−1.15	−3.64	1.35	2.31%	−2.84*	0.81	−1.50	3.12	0.77%	−5.15**	−2.72	−5.84	0.40	3.13%
COVID diagnosis (ref = no diagnosis)	−2.31	−3.18	−10.77	4.41	0.35%	3.29	2.62	−4.42	9.66	0.39%	−0.67	−1.22	−9.88	7.43	0.03%
Outdoors’s time	4.73***	1.64	−0.59	3.87	5.26%	5.21***	2.92*	0.70	5.15	8.01%	4.79***	0.83	−1.77	3.43	3.63%
Stress from lockdown restrictions	−3.68	−0.28	−3.60	3.03	0.86%	−2.85	0.02	−2.83	2.86	0.49%	−3.91	−0.66	−4.31	2.99	0.80%
Relationship stress	−5.99***	−2.54	−5.29	0.21	4.80%	−4.61**	−1.91	−4.77	0.95	2.74%	−6.15***	−1.31	−4.78	2.17	3.29%
Changes in relationship quality	7.35**	4.5*	1.19	7.81	4.82%	7.55**	5.23*	1.29	9.17	5.75%	7.3**	3.64	−0.52	7.80	3.12%

* = *p* < 0.05, ** = *p* < 0.01, *** = *p* < 0.001, LL = lower limit of bootstrapped 95%CI, UL = upper limit of bootstrapped 95%CI.

**Table 5:** Results of the unadjusted (univariate) and adjusted (multivariate) regressions for achieving in life, personal relationships, and personal safety domains.

	Achieving in life					Personal relationships					Personal safety				
	Unadjusted	Adjusted				Unadjusted	Adjusted				Unadjusted	Adjusted			
	B	B	LL	UL	*R*^2^	B	B	LL	UL	*R*^2^	B	B	LL	UL	*R*^2^
Age	−0.01	−0.01	−0.30	0.28	0.02%	0.07	0.07	−0.26	0.41	0.12%	−0.01	−0.01	−0.31	0.28	0.02%
Gender (ref = male)	−9.04*	−9.04*	−16.80	−1.28	1.65%	−1.21	−1.21	−13.30	10.88	0.02%	−2.47	−2.47	−10.89	5.94	0.11%
Pre-COVID mental health	8.16***	8.04***	4.55	11.53	12.73%	6.36**	6.65**	2.35	10.95	6.47%	6.97***	7.02***	3.03	11.00	8.80%
Physical health	9.5***	5.98**	2.41	9.55	10.71%	11.17***	10.33***	5.26	15.39	15.12%	9.15***	6.86***	2.74	10.97	10.23%
**Occupation (ref = nurses)**															
Allied health	6.92	7.48	−0.18	15.14	3.65%	3.32	3.73	−5.00	12.46	0.40%	6.67	6.9	−1.93	15.74	1.42%
Non-med	4.39	4.13	−9.18	17.43	3.65%	4.12	3.82	−13.04	20.68	0.40%	−1.64	−1.76	−16.57	13.05	1.42%
Physicians	13.72**	11.37*	1.94	20.80	3.65%	−0.54	−1.2	−13.82	11.43	0.40%	4.81	3.99	−7.92	15.91	1.42%
Supervisor support	3.48**	2.67*	0.48	4.85	3.41%	2.81*	2.52	−0.24	5.28	2.00%	3.03*	2.62*	0.05	5.19	2.67%
Direct care (ref = no direct care)	−3	−2.35	−9.00	4.30	0.39%	−4.06	−3.62	−11.36	4.12	0.61%	−2.21	−1.96	−9.24	5.31	0.21%
Work infection risk	−3.77*	−2.03	−5.05	0.99	1.92%	−2.17	−0.88	−4.28	2.51	0.40%	−4.76**	−3.61*	−7.06	−0.17	3.61%
Community infection risk	−0.62	−1.76	−5.25	1.72	0.43%	−3.05	−3.8	−7.65	0.04	2.10%	−1.92	−3.08	−6.79	0.63	1.35%
COVID-19 infection worries	−4.77**	−1.93	−5.12	1.26	2.36%	−2.63	0.67	−3.22	4.55	0.35%	−6.22***	−3.66	−7.59	0.26	4.58%
COVID diagnosis (ref = no diagnosis)	−1.28	−2.52	−11.02	5.99	0.12%	−2.53	−3.2	−12.89	6.48	0.21%	−3.15	−4.03	−13.93	5.87	0.38%
Outdoors’s time	4.48**	1.87	−1.03	4.77	3.46%	6.24***	3.38*	0.25	6.51	6.21%	3.51*	0.03	−2.98	3.04	1.45%
Stress from lockdown restrictions	−4.62	−1.27	−5.59	3.05	1.21%	−1.44	1.27	−3.14	5.67	0.12%	−4.3	−0.22	−4.83	4.38	0.81%
Relationship stress	−5.8***	−2.55	−6.12	1.01	3.23%	−6.14**	−2.5	−6.43	1.42	2.93%	−6.24**	−2.87	−6.95	1.21	3.59%
Changes in relationship quality	6.68*	4.55	0.11	9.00	3.01%	11.06***	7.96**	3.15	12.77	7.02%	5.96*	3.32	−1.33	7.97	1.93%

* = *p* < 0.05, ** = *p* < 0.01, *** = *p* < 0.001, LL = lower limit of bootstrapped 95%CI, UL = upper limit of bootstrapped 95%CI.

**Table 6:** Results of the unadjusted (univariate) and adjusted (multivariate) regressions for Community Connectedness and Future Security domains.

	Community connectedness					Future Security				
	Unadjusted	Adjusted				Unadjusted	Adjusted			
	B	B	LL	UL	*R*^2^	B	B	LL	UL	*R*^2^
Age	0.18	0.18	−0.13	0.49	0.70%	0.1	0.1	−0.22	0.43	0.23%
Gender (ref = male)	−5.72	−5.72	−14.05	2.62	0.51%	−7.1	−7.1	−16.16	1.97	0.83%
Pre-COVID mental health	5.41**	5.45**	1.47	9.43	4.47%	6.03**	5.75**	0.76	10.75	5.51%
Physical health	10.09***	8.42**	3.28	13.56	11.22%	10.65***	8.45***	3.48	13.41	12.50%
**Occupation (ref = nurses)**										
Allied health	6.53	7.52	−2.57	17.60	1.55%	9.93*	10.76*	1.67	19.84	5.00%
Non-med	2.75	1.89	−13.47	17.25	1.55%	−5	−5.68	−20.84	9.48	5.00%
Physicians	7.86	6.69	−6.67	20.04	1.55%	14.33*	13.16	0.01	26.31	5.00%
Supervisor support	5.04***	4.65**	1.85	7.45	6.57%	5.76***	5.09***	2.22	7.95	8.66%
Direct care (ref = no direct care)	−2.23	−1.24	−8.90	6.42	0.13%	−2.64	−2.09	−9.93	5.75	0.25%
Work infection risk	−4.74*	−3.05	−6.83	0.74	2.59%	−5.83**	−3.74*	−7.26	−0.21	4.17%
Community infection risk	−1.98	−1.92	−6.01	2.18	0.74%	−1.98	−2.13	−5.55	1.28	0.85%
COVID-19 infection worries	−3.9*	−0.2	−4.00	3.59	0.86%	−4.66**	−0.97	−4.53	2.58	1.47%
COVID diagnosis (ref = no diagnosis)	−3.32	−4.07	−12.98	4.84	0.35%	−8.5	−9.81	−19.51	−0.12	2.18%
Outdoors’s time	4.07*	0.92	−2.17	4.01	2.01%	4.82**	1.55	−1.42	4.51	3.14%
Stress from lockdown restrictions	−4.16	−0.91	−5.93	4.12	0.71%	−4.47	−0.19	−4.77	4.39	0.74%
Relationship stress	−8.09***	−5.48*	−9.92	−1.05	6.39%	−4.94*	−1.15	−5.32	3.01	1.47%
Changes in relationship quality	8.14**	5.43*	0.85	10.00	3.51%	4.75	1.4	−3.06	5.86	0.82%

* = *p* < 0.05, ** = *p* < 0.01, *** = *p* < 0.001, LL = lower limit of bootstrapped 95%CI, UL = upper limit of bootstrapped 95%CI.

**Figure 3:**
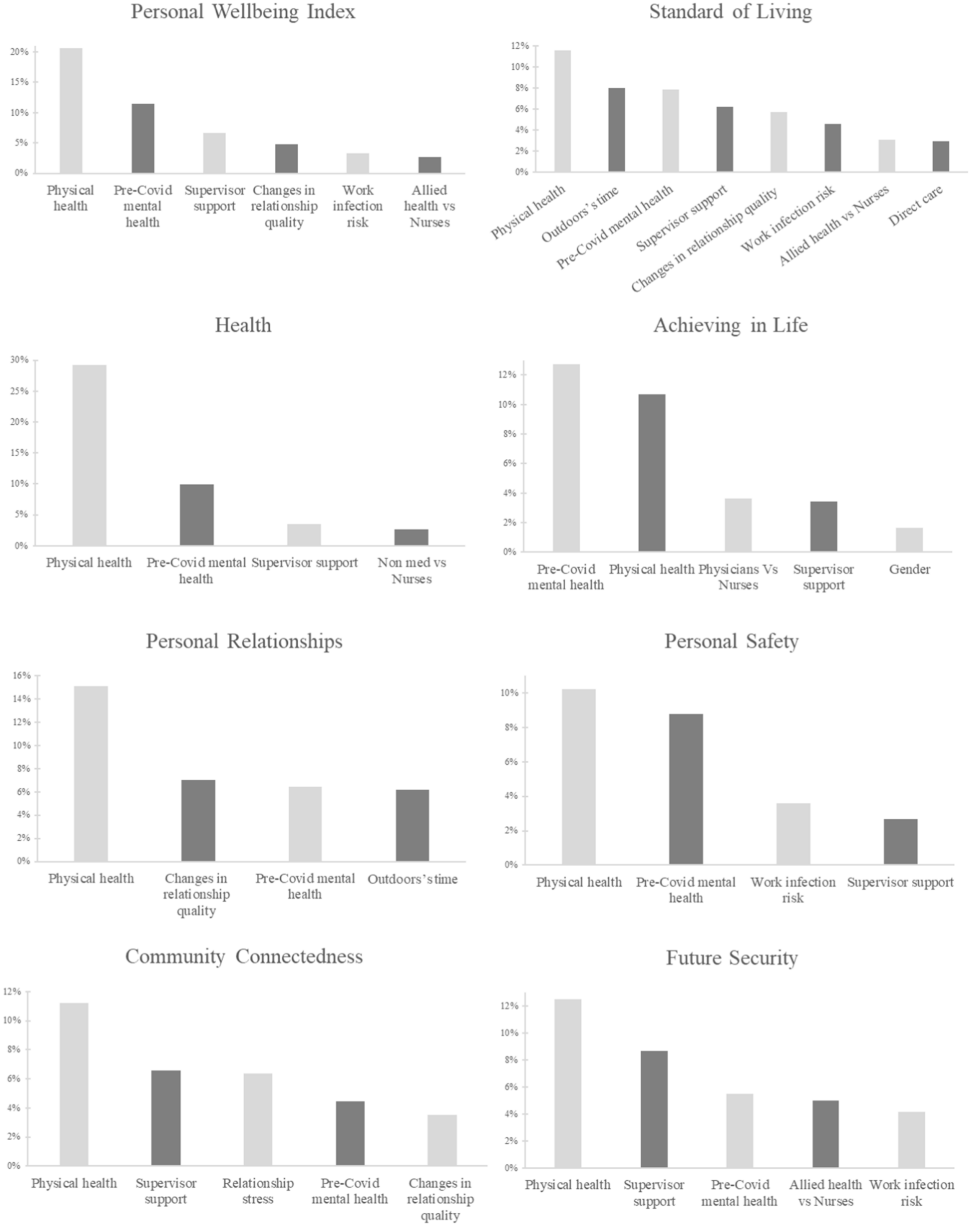
Relative importance analysis-subjective wellbeing and sub-domains.

Based on the relative importance analysis, physical health had a large effect ( $R^{2}$ = 20.60%) and explained the most variance in overall subjective wellbeing. The other significant predictors explained moderate amounts of variance in subjective wellbeing, ranging from 2.67 to 11.41% in $R^{2}$ . Physical health also consistently explained the most variance in all domains, except for achieving in life, where pre-COVID-mental health explained the most variance. Ranking of predictors variance explained in overall subjective wellbeing and in its sub-domains are shown in Figure 3.

## Discussion

To the authors knowledge, this study is one of the first Australian, and one of few studies globally, to investigate COVID-19’s mental health and wellbeing impacts on FHWs in 2022 during the Omicron wave. Specifically, the current study investigated the predictors of psychological distress and subjective wellbeing in Victorian (Australia) FHWs during the Omicron wave in January 2022. When compared to population norms assessed during the same time (Biddle and Gray, 2022; Khor et al., 2022), sample means in this study showed significantly higher psychological distress and lower wellbeing. Findings also identified multifactorial predictors of FHWs’ psychological distress and wellbeing during the Omicron wave, which included COVID infection related factors, age, health factors, relational factors, and supervisor support.

Consistent with previous findings, this study affirms the need for interventions targeting infection related factors during the COVID-19 pandemic and future infectious disease outbreaks. Specifically, infection risks and COVID-19 diagnosis were found to be predictive of psychological distress, with COVID-19 infection worries identified as the strongest independent risk factor. Notably, the effect of COVID-19 infection worries on FHWs’ psychological distress was found to be independent of perceived risk of infection or a COVID-19 diagnosis. This suggest that psychological distress associated with COVID-19 worries may persist at high levels among FHWs even when risk of infection is minimal. This likely explains why despite improvements in infection control procedures, increased vaccination uptake and an overall decrease in infection rates (Braun et al., 2021; Damluji et al., 2021; Dunbar et al., 2021), there continues to be persisting anxiety around COVID-19 infections among FHWs (Hendricksen et al., 2022; Feng et al., 2023; Scott et al., 2023). It appears that more needs to be done to manage FHWs’ concerns and anxieties with being infected, which seems to be wide-ranging. Studies suggest that, in addition to health concerns, FHWs also have significant worries around the social impacts of COVID-19 infections, such as social isolation, infecting vulnerable family members, disrupted family routines and for some, being stigmatised after infection (Schaffer et al., 2022; Sheen et al., 2022). These findings highlight the need to go beyond infection control when attempting to reduce anxiety in FHWs around COVID-19 infections, such as integrating supports focused on mitigating the social impacts of being infected, as well as psychological interventions that target FHWs’ anxiety management around COVID.

In this study, younger FHWs have also been found to be an at-risk group for higher levels of psychological distress, consistent with previous findings on FHWs (Kisely et al., 2020; Moitra et al., 2021; Czepiel et al., 2022), as well as on the general public (Xiong et al., 2020; Dragioti et al., 2022). With regards to younger FHWs, it has been suggested that their vulnerability to psychological distress may be due to their lack of specialised experience and training (Lee et al., 2021; Van Wert et al., 2022), which is not only essential for infection control, but to also protect against mental health issues as they can likely bolster confidence at work and mitigate infection related anxieties (De Brier et al., 2020; Kisely et al., 2020). Additionally, given the high rates of psychological distress in the general public (Xiong et al., 2020; Dragioti et al., 2022), it is important to consider the social impacts of the pandemic on younger FHWs. Studies on the general public have found concerning levels of loneliness among younger adults that have led to increased distress (Li and Wang, 2020). It is likely that as emerging adults, they have yet to develop strong social connections and supports, which are important to protect against mental health impacts during traumatic events such as the COVID-19 pandemic (McGuire et al., 2018; Kaniasty et al., 2020). This is significant as social support has been implicated to play an important role in FHWs’ mental health resilience, especially in younger FHWs (Hou et al., 2020). Nevertheless, it is evident that younger FHWs are in need of targeted interventions and, given recent findings, it appears that social support interventions may be beneficial, especially when tailored to enhance their confidence at work, improve work related stress management and increase their social connectedness and support at work (Mohamed et al., 2022; Musgrove et al., 2022).

With regards to wellbeing, health factors have emerged as the strongest predictor. Specifically, better physical health and mental health status were found to be highly protective of lower wellbeing, which also extended to psychological distress, however, to a lower extent. This indicates that better health states can potentially buffer COVID-19 impacts on FHWs’ mental health, as well as their wellbeing across multiple life domains. Wellbeing findings in particular, are noteworthy as the few studies that have considered it confirms that FHWs’ wellbeing has been disproportionately affected compared to the general public during the COVID-19 pandemic (McFadden et al., 2021; McGuinness et al., 2022). Adding to this, the current study found that wellbeing impacts on FHWs were evident across a wide range of life domains, including their health, relationships, community connectedness, future security, life achievements and safety. To date wellbeing outcomes in FHWs have been largely overlooked and many may conflate wellbeing and mental health outcomes together. However, this study shows that wellbeing intervention targets needs to be considered on its own as key predictors identified for wellbeing were different to those for psychological distress. Health status as a key predictor suggest that wellbeing interventions may require more long-term approaches that maintain optimal physical and mental health. This could involve targeting persisting issues that have been documented to affect health statuses in FHWs regardless of infectious disease outbreaks, such as burnout and excessive workloads (Kim et al., 2011; Adriaenssens et al., 2015; Salvagioni et al., 2017; Verougstraete and Hachimi Idrissi, 2020).

This study has also found relational factors as important indicators of psychological distress and wellbeing among FHWs. We found that greater familial and social relationship stress was a risk factor for psychological distress, while positive changes to these relationships were protective of lower wellbeing. These findings further support the notion that social factors play a critical role in FHWs’ mental health and wellbeing (Lim et al., 2010; McKinley et al., 2019). It highlights the importance of not only enhancing social relationships but also safeguarding it for FHWs during times of crisis. This is important because it is well documented that, while FHWs have poor help seeking behaviour with regards to mental health (Halter Margaret, 2004; Brooks et al., 2011; Galbraith et al., 2014; Wijeratne et al., 2021), they rely heavily on social support to manage it (Labrague, 2021; Schug et al., 2021). Thus, during times when widespread stigma and social rejection of FHWs are common, such as infectious disease outbreaks (Gómez-Durán et al., 2020; Schubert et al., 2021; Yuan et al., 2021; Ding et al., 2022), they can easily be left isolated and more vulnerable to mental health and wellbeing issues. Moreover, healthcare work during this time may have also placed additional stressors on FHWs’ social contacts and personal relationships, such as increased familial anxiety due to infection risks, poor work-life balance and stigma as a FHW family (Ali et al., 2020; Evanoff et al., 2020; Schaffer et al., 2022; Sheen et al., 2022). FHWs are currently experiencing tremendous challenges, and these findings underscore the importance of protecting FHWs social relationships, which can have multi-fold effects on their mental health and wellbeing.

Lastly, another key predictor of both mental health and wellbeing in FHWs to consider is supervisor support. In line with previous studies (Evanoff et al., 2020; Feingold et al., 2021; Greco et al., 2022), findings show that support from supervisors during the pandemic can play an important role in influencing FHWs mental health and wellbeing. This echoes the call for increased focus on supervisors’ capabilities with regards to supporting FHWs’ mental health and wellbeing (Carmassi et al., 2020; Hennein et al., 2021). While providing extensive mental health support may be out of scope for supervisors, they are still in unique positions to provide a range of social, work, and emotional support directly to FHWs that can influence their mental health and wellbeing. For example, Evanoff et al. (2020) found that family specific supervisory support was strongly associated with better mental health and wellbeing among FHWs. Another study also found that ethical leadership from supervisors was significantly associated with lower levels of work-related stress, which includes promoting and modelling openness, integrity, and trustworthiness (Zhou et al., 2015). It is also important to note that supervisors themselves have been experiencing additional stress and psychological burden beyond those experienced by their staff during the pandemic (Middleton et al., 2021), likely due to the additional support they are required to provide their staff. Thus, it follows that to ensure organisational support for FHWs are effectively implemented and managed, organisations need to consider strategies to elevate the additional burden on supervisors during this time. Nevertheless, supervisor support is likely an important pathway for organisations to influence FHWs mental health and wellbeing, and therefore should be a core focus in organisational mental health and wellbeing strategies.

### Limitations

When interpreting findings in this study, several limitations should be considered. Firstly, as a cross-sectional study, it is not evident that the distress observed in this study is indicative of an acute reaction or persisting distress, which should be investigated further in longitudinal studies. Additionally, causal links between variables should be interpreted with caution. While the use of DAGs in this study provides a framework around causality between investigated variables, it relies on the assumptions in the DAGs. Other models may exist and the model in this study is not intended to be a proposal for a theoretical framework around FHWs’ mental health and wellbeing. The use of the DAG in this study is intended to be a way to systematically adjust for covariates to estimate effects and provide transparency around assumed relationships (Ferguson et al., 2020). Secondly, mediation analysis was beyond the scope of this study, however, the results points to several mediating relationships among the investigated variables and should be considered when interpreting results and in future research. It is thus recommended that future research investigate these mediating relationships further through structural equation modelling or mediation analyses. Thirdly; there was a low representation of physicians, which may have impacted the generalisability of results for this cohort. Lastly, due to the small sample size, precision of estimates may be weak, thus effects and mean differences with population norms should be interpreted and generalised with caution.

## Conclusion

In sum, the COVID-19 pandemic continues to place undue pressure on FHWs’ mental health and wellbeing. Findings indicate that FHWs mental health and wellbeing are associated with a wide range of factors that includes work-related and social determinants. It is thus important to consider a wide range of factors, including those beyond work, when developing targeted interventions and support for FHWs’ mental health and wellbeing, to ensure their effectiveness. Nevertheless, findings reinforce the need for ongoing research, development, and implementation of targeted interventions for FHWs who continue to face significant challenges.

## Data availability statement

The datasets presented in this article are not readily available because the datasets investigated and presented in this study are not available for public use as ethical approvals for this study does not include public availability of participants data. Requests to access the datasets should be directed to brian.lee@deakin.edu.au.

## Ethics statement

The studies involving human participants were reviewed and approved by Deakin University High Risk Ethics Committee and Eastern Health’s Ethics Committee. The patients/participants provided their written informed consent to participate in this study.

## Author contributions

BL, ML, LB, CAO, and JS was involved in the conceptualisation and design of this study. BL was responsible for the formal analysis, data curation, and investigation under the supervision of ML, LB, CAO, and JS. BL prepared the original manuscript draft, which was then review and edited by ML, LB, CAO and JS. All authors contributed to the article and approved the submitted version.

## Funding

Preparation of this paper was supported by using award money from the Victorian COVID-19 Research Fund-Stream B, State Government of Victoria. The funders of this study had no role in study design, data collection and analysis, decision to publish, or preparation of the manuscript. CAO is supported by a NHMRC Investigator Grant GNT1175086.

## Conflict of interest

The authors declare that the research was conducted in the absence of any commercial or financial relationships that could be construed as a potential conflict of interest.

## Publisher’s note

All claims expressed in this article are solely those of the authors and do not necessarily represent those of their affiliated organizations, or those of the publisher, the editors and the reviewers. Any product that may be evaluated in this article, or claim that may be made by its manufacturer, is not guaranteed or endorsed by the publisher.

## Footnotes

^1^https://www.dagitty.net/
