# Supplementary material for: Key predictors of psychological distress and wellbeing in Australian frontline healthcare workers during COVID-19 (Omicron wave)
Source: Front Psychol. 2023 Jul 7;14:1200839. doi: 10.3389/fpsyg.2023.1200839 (PMC10361570; doi:10.3389/fpsyg.2023.1200839)
Supplement: Supplementary file 2 [file Table_2.docx]

Supplementary Material

**Key Predictors of Psychological Distress and Wellbeing in Frontline Healthcare Workers during COVID-19**

$\mathbf{Brian En Chyi Lee}^{\boldsymbol{1}}$**,** $\mathbf{Mathew Ling}^{\boldsymbol{1,2}}$**,** $\mathbf{Leanne Boyd}^{\boldsymbol{3}}$**,** $\mathbf{Craig Olsson}^{\boldsymbol{1}}\boldsymbol{,}\mathbf{Jade Sheen}^{\boldsymbol{1}}$

*** Correspondence:**

Email address: [brian.lee@deakin.edu.au](mailto:brian.lee@deakin.edu.au) (Brian En Chyi Lee)

Supplementary Figure 1. Causal DAG for variables used in multivariate regressions for psychological distress (K6) and subjective wellbeing (SWI)


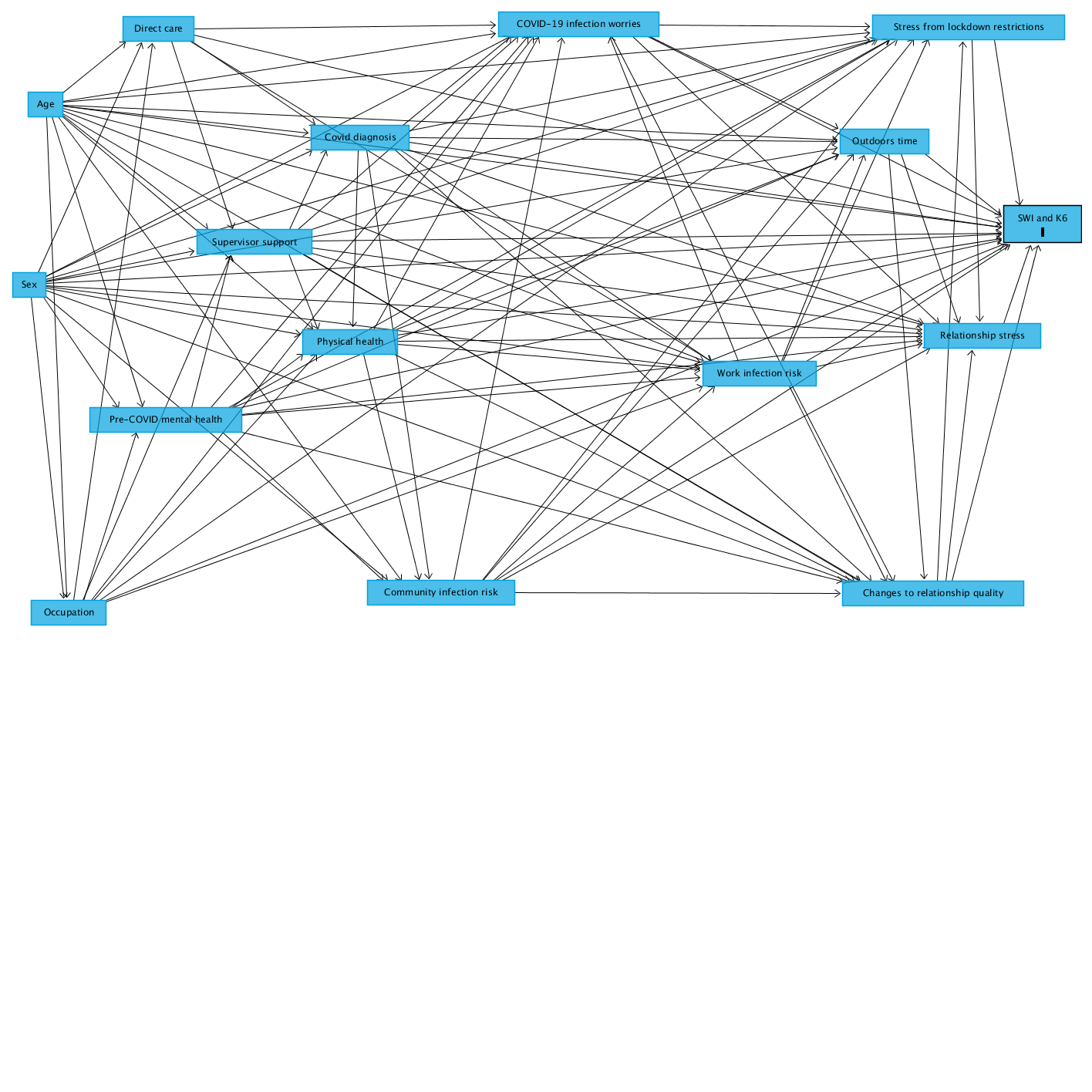


***Causal relationships between variables expressed in the DAG***

"COVID-19 infection worries" -> "Changes to relationship quality"

"COVID-19 infection worries" -> "Outdoors time"

"COVID-19 infection worries" -> "Relationship stress"

"COVID-19 infection worries" -> "SWI and K6"

"COVID-19 infection worries" -> "Stress from lockdown restrictions"

"Changes to relationship quality" -> "Relationship stress"

"Changes to relationship quality" -> "SWI and K6"

"Changes to relationship quality" -> "Stress from lockdown restrictions"

"Community infection risk" -> "COVID-19 infection worries"

"Community infection risk" -> "Changes to relationship quality"

"Community infection risk" -> "Outdoors time"

"Community infection risk" -> "Relationship stress"

"Community infection risk" -> "SWI and K6"

"Community infection risk" -> "Stress from lockdown restrictions"

"Community infection risk" -> "Work infection risk"

"Covid diagnosis" -> "COVID-19 infection worries"

"Covid diagnosis" -> "Changes to relationship quality"

"Covid diagnosis" -> "Community infection risk"

"Covid diagnosis" -> "Outdoors time"

"Covid diagnosis" -> "Physical health"

"Covid diagnosis" -> "Relationship stress"

"Covid diagnosis" -> "SWI and K6"

"Covid diagnosis" -> "Stress from lockdown restrictions"

"Covid diagnosis" -> "Work infection risk"

"Direct care" -> "COVID-19 infection worries"

"Direct care" -> "Covid diagnosis"

"Direct care" -> "SWI and K6"

"Direct care" -> "Supervisor support"

"Direct care" -> "Work infection risk"

"Outdoors time" -> "Changes to relationship quality"

"Outdoors time" -> "Relationship stress"

"Outdoors time" -> "SWI and K6"

"Physical health" -> "COVID-19 infection worries"

"Physical health" -> "Changes to relationship quality"

"Physical health" -> "Community infection risk"

"Physical health" -> "Outdoors time"

"Physical health" -> "Relationship stress"

"Physical health" -> "SWI and K6"

"Physical health" -> "Stress from lockdown restrictions"

"Physical health" -> "Work infection risk"

"Pre-COVID mental health" -> "COVID-19 infection worries"

"Pre-COVID mental health" -> "Changes to relationship quality"

"Pre-COVID mental health" -> "Community infection risk"

"Pre-COVID mental health" -> "Outdoors time"

"Pre-COVID mental health" -> "Physical health"

"Pre-COVID mental health" -> "Relationship stress"

"Pre-COVID mental health" -> "SWI and K6"

"Pre-COVID mental health" -> "Stress from lockdown restrictions"

"Pre-COVID mental health" -> "Supervisor support"

"Pre-COVID mental health" -> "Work infection risk"

"Relationship stress" -> "SWI and K6"

"Stress from lockdown restrictions" -> "Relationship stress"

"Stress from lockdown restrictions" -> "SWI and K6"

"Supervisor support" -> "COVID-19 infection worries"

"Supervisor support" -> "Changes to relationship quality"

"Supervisor support" -> "Covid diagnosis"

"Supervisor support" -> "Physical health"

"Supervisor support" -> "Relationship stress"

"Supervisor support" -> "SWI and K6"

"Supervisor support" -> "Stress from lockdown restrictions"

"Supervisor support" -> "Work infection risk"

"Work infection risk" -> "COVID-19 infection worries"

"Work infection risk" -> "Changes to relationship quality"

"Work infection risk" -> "Outdoors time"

"Work infection risk" -> "Relationship stress"

"Work infection risk" -> "SWI and K6"

"Work infection risk" -> "Stress from lockdown restrictions"

Age -> "COVID-19 infection worries"

Age -> "Changes to relationship quality"

Age -> "Community infection risk"

Age -> "Covid diagnosis"

Age -> "Direct care"

Age -> "Outdoors time"

Age -> "Physical health"

Age -> "Pre-COVID mental health"

Age -> "Relationship stress"

Age -> "SWI and K6"

Age -> "Stress from lockdown restrictions"

Age -> "Supervisor support"

Age -> "Work infection risk"

Age -> Occupation

Occupation -> "COVID-19 infection worries"

Occupation -> "Direct care"

Occupation -> "Physical health"

Occupation -> "Pre-COVID mental health"

Occupation -> "SWI and K6"

Occupation -> "Stress from lockdown restrictions"

Occupation -> "Supervisor support"

Occupation -> "Work infection risk"

Sex -> "COVID-19 infection worries"

Sex -> "Changes to relationship quality"

Sex -> "Community infection risk"

Sex -> "Covid diagnosis"

Sex -> "Direct care"

Sex -> "Outdoors time"

Sex -> "Physical health"

Sex -> "Pre-COVID mental health"

Sex -> "Relationship stress"

Sex -> "SWI and K6"

Sex -> "Stress from lockdown restrictions"

Sex -> "Supervisor support"

Sex -> "Work infection risk"

Sex -> Occupation

**
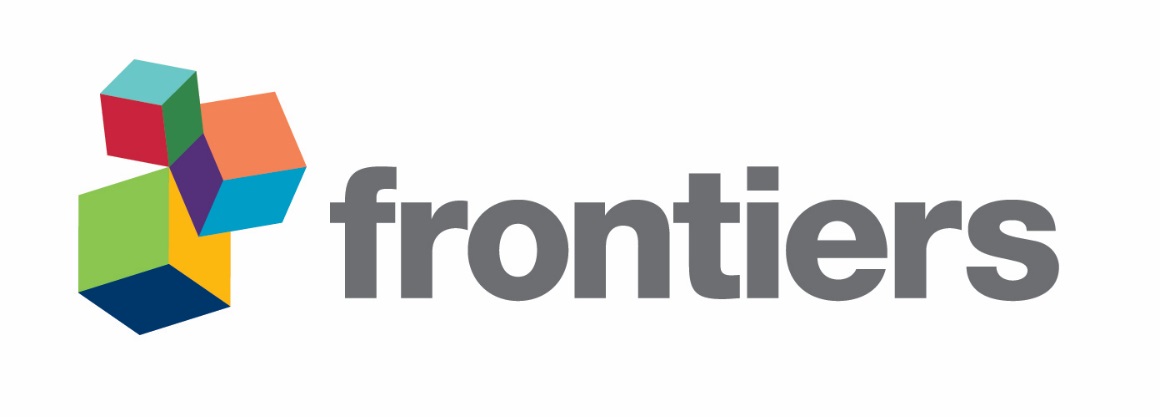
**
